# Coordination of cortex modifications in time, space, and under stress

**DOI:** 10.1111/nph.70581

**Published:** 2025-09-19

**Authors:** Dorota Kawa, Hannah M. Schneider, Kaisa Kajala

**Affiliations:** ^1^ Experimental & Computational Plant Development, Department of Biology, Institute of Environmental Biology Utrecht University Padualaan 8 3584CH Utrecht the Netherlands; ^2^ Plant Stress Resilience, Department of Biology, Institute of Environmental Biology Utrecht University Padualaan 8 3584CH Utrecht the Netherlands; ^3^ Leibniz Institute of Plant Genetics & Crop Plant Research (IPK) OT Gatersleben Corrensstr 3 06466 Seeland Germany; ^4^ Division of Root Sciences, Department of Crop Science Georg‐August‐University Goettingen Goettingen Germany

**Keywords:** aerenchyma, cell‐type identity, cell‐type plasticity, cortex, endodermis, exodermis, MCS, symbiotic interactions

## Abstract

In roots, cell‐type‐specific differentiation enables specialized responses to environmental stress. The cortex, located between the vasculature and epidermis, is a key site for stress‐responsive modifications. The distinct specializations of the cortex are controlled by developmental, positional and environmental signals. Cortex layers are developmentally and transcriptionally diverse, with capacities of forming protective barriers such as endodermis and exodermis and other cell‐type modifications such as multiseriate cortical sclerenchyma and aerenchyma to aid in edaphic stress tolerance. Additionally, the cortex is essential in forming nitrogen‐fixing nodules and arbuscules, and therefore symbiotic interactions. These modifications enhance stress resilience by regulating the two‐way fluxes of water, solutes and nutrients between the soil and the plant, increasing mechanical strength or facilitating biotic interactions. Understanding how cortex modifications coexist, synergize to influence plant fitness, or compensate for each other remains a challenge. Future research should focus on their combined effects across root types to reveal trade‐offs and optimize stress protection.

**Table:** 

	Contents	
	Summary	1677
I.	Introduction	1677
II.	Ground zero: the cortex	1678
III.	Solid ground: forming cortex layers and their specific capacities	1678
IV.	Shifting ground: cortex differentiation trajectories and their plasticity	1678
V.	Common ground: cortex layer capacities for symbiotic interactions	1682
VI.	Breaking ground: future directions to utilize cortex potential	1682
VII.	Conclusions	1683
	Acknowledgements	1683
	References	1683

## I. Introduction

In multicellular organisms, the specialization of distinct cell types forms the foundation of their structural and functional complexity and enables efficient responses to diverse environmental challenges. In plants, the launching of stress‐responsive pathways is often balanced with the maintenance of cell‐type identity (Iyer‐Pascuzzi *et al*., 2011; Rich‐Griffin *et al*., 2020), or, in some cases, the environmental signals can induce differentiation or even reprogramming into different cell types, changing the proportions and patterning of cell types in an organ (Shulse *et al*., 2019; Wang *et al*., 2021). In the root, several cell‐type‐specific responses to environmental fluctuations affect the cells' morphology and are driven by turning on their differentiation processes.

## II. Ground zero: the cortex

**Fig. 1:**
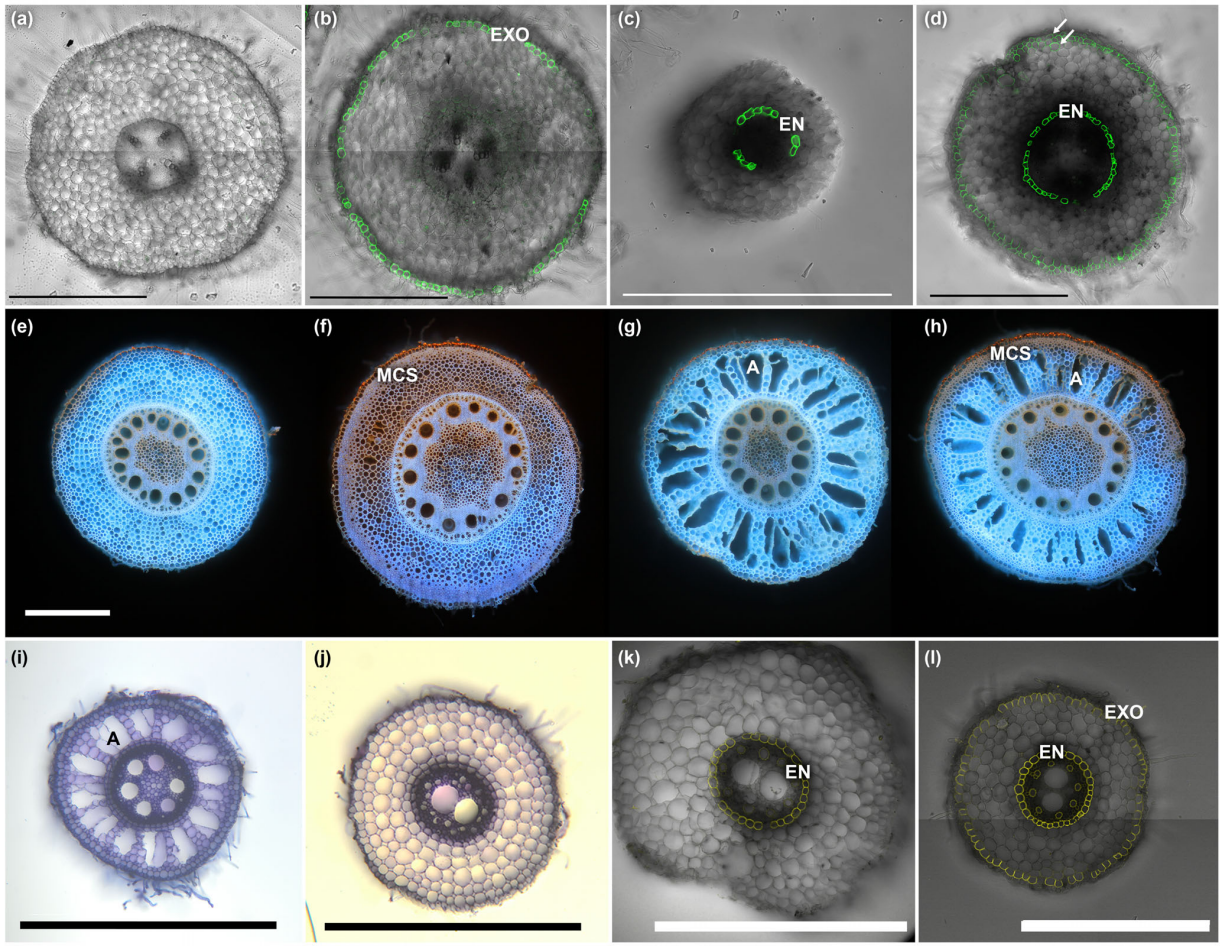
Diversity in cortex layer number and morphology. The legumes (a–d) and grasses (e–l) can have high numbers of cortex layers, which also can vary within the species depending on the root type, developmental stage and environment. The cortex layers display a diversity of modifications, here shown as examples of exodermal suberin (b, l), endodermal suberin (c, d, k, l), multiseriate cortical sclerenchyma (f, h) and aerenchyma (g, h, i), that also are regulated by developmental stage, environment and root type. (a–d) Legume primary root cross sections stained with fluorol yellow 088, a stain for suberin and lipids (Lux *et al*., 2005), show the plasticity of exodermis (EXO) and endodermis (EN) suberization. In chickpea (*Cicer arietinum*) seedlings, no suberin is detected in control conditions (a) and the exodermis becomes suberized in response to abscisic acid (ABA) (b). In *Medicago truncatula* (c) and *Vicia sativa* (d) seedlings, the endodermis becomes suberized in response to ABA. In *Vicia sativa*, additional fluorol yellow signal is detected in the outermost cortex layers (white arrows), but this does not correspond with lamellar suberin (Jo *et al*., 2025b). (a–d) Eight‐day‐old seedlings were grown on MS plates, with 72‐h treatment with mock (a) or 2 μM ABA (b–d) treatment (Jo *et al*., 2025b). Bars, 0.5 mm. Images courtesy of Leonardo Jo. (e–h) Maize (*Zea mays*) nodal roots from a diversity panel show diversity also in cortex modifications, including multiseriate cortical sclerenchyma (MCS) in the outermost cortex layers and aerenchyma (A) throughout the cortex layers. When MCS and aerenchyma are seen in the same root, aerenchyma is excluded from the outermost cortex layers. Cross sections from the third stem node were collected at anthesis at 5–8 cm from the root–shoot junction and imaged by their autofluorescence in UV light. Bars, 0.5 mm. (i–l) Crown (i) and seminal (j) roots of sorghum (*Sorghum bicolor*) differ in the number of cortex layers and capacity to form aerenchyma. Suberin deposition patterns differ along the primary root axis, starting with suberin only in the endodermis (k) and further from the root tip in the endodermis and exodermis (l). Cross sections collected mid region of 28‐d‐old plant (i, j) or from primary root regions proximal (k) and distant (l) from the root tip were stained with toluidine blue (i, j) and fluorol yellow 088 (k, l). Bars, 0.5 mm. Images (k, l) courtesy of Julia Mars.

**Fig. 2:**
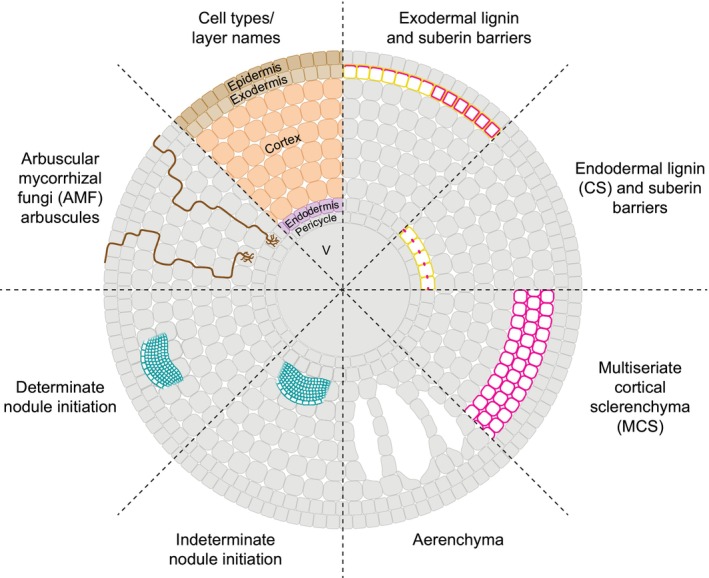
Cortex modifications discussed in this article and their relative positions across the cortex layers. CS, Casparian strip.

The cortex stands out in the root cellular landscape for stress responses due to the remarkable diversity of form and functions resulting from differentiation processes. In this Tansley insight, we consider cortex in the broader anatomical sense, in other words, as a heterogeneous set of cells located between root vasculature and epidermis, often synonymously known as the root ground tissue. This definition for cortex is wider than that typically used in root developmental biology, where ‘cortex’ refers to the structurally undifferentiated cortex parenchyma. The strict definition served well in Arabidopsis, where young root tip contains only a single layer of cortex, accompanied by an additional middle cortex layer upon juvenile‐to‐adult phase transition (Baum *et al*., 2002). However, many important crop model species such as grasses or legumes have roots with several cortex layers (Fig. 1). These layers have different capacities to launch differentiation trajectories to develop diverse cellular morphologies and tissue functions (here termed ‘cortex modifications’), as exemplified noncomprehensively in Fig. 2 and Box 1. Excitingly, these responses and trajectories often are mounted in response to abiotic and biotic stresses to form different stress‐protective tissues.

Box 1 **Glossary**

**Table:** 

Cortex	A heterogeneous set of cells located between root vasculature and epidermis. Also known as the root ground tissue.
Cortical parenchyma	The cortical cell ‘type’ that has not undergone any further cell wall development, specialized cell divisions, senescence or apoptosis. They are often characterized by their relatively thin primary cell wall made of cellulose, pectin and hemicellulose and large intercellular spaces between them.
*Cortical modifications*	
Cortical modification	Any structural, anatomical or cellular change in the cortex of a root that alters its form or function.
Endodermis	Innermost cortical layer that deposit lignin and suberin in their cell walls to provide apoplastic barrier functions. Present in all vascular plants. Typically uniseriate, but also biseriate endodermis exists in horsetails (Bower, 1935). For a recent review, see Andersen & Drapek (2024).
Aerenchyma	Tissue containing enlarged gas spaces, exceeding in size those commonly found as intracellular spaces.
Multiseriate cortical sclerenchyma	Multiple layers of small cells with thick, lignin‐encrusted cell walls located in the outer cortex of nodal roots (i.e. a sub‐type of sclerenchyma, defined by its position in the cortex and small cells).
Hypodermis	The subepidermal cell layer(s) of unspecialized or specialized cortical parenchyma.
Exodermis	Outermost cortical layer(s) that can deposit suberin and/or lignin in their cell walls to provide apoplastic barrier functions. Lignin deposition can be anticlinal (‘Casparian strip’), polar cap (soil‐facing) or nonpolar. Exodermis differentiation is often induced by environmental cues. Typically uniseriate, but multiseriate exodermis is common in certain lineages (Perumalla *et al*., 1990; Peterson & Perumalla, 1990). For recent reviews on exodermis, see Liu & Kreszies (2023), Jones *et al*., (2025), Cantó‐Pastor *et al*. (2025).
Nodules	Specialized structures that form on the roots as a result of a symbiotic relationship with nitrogen‐fixing bacteria. Determinate nodules are spherical structures that stop growing after formation, lack a persistent meristem and contain cells at a similar developmental stage. Indeterminate nodules are elongated, maintain a persistent meristem for continuous growth and exhibit distinct developmental zones from tip to base. Diversity of forms recently reviewed in Kundu *et al*. (2025) and Schiessl & Jhu (2025).

Here, we also consider endodermis as one of the cortex modifications, because it is specified from the same initial cell as cortex parenchyma (see Section III). Moreover, in many species endodermis shares morphological, functional and genetic similarities with the outermost cortex layer, exodermis. Furthermore, as increasing amounts of root research is carried out in diverse model systems, we hope to use the terminology traditionally applied in these systems (Esau, 1967; Lux *et al*., 2004), that is, cortex is a composite layer of tissues located between vasculature and epidermis.

In this Tansley insight, we discuss how the cortex coordinates multiple stress‐responsive differentiation trajectories that are also regulated developmentally and spatially. While a lot remains to be discovered, we also propose that a shift toward studying all cortical cell trajectories together is pivotal for our understanding of their functionality in stress protection.

## III. Solid ground: forming cortex layers and their specific capacities

The cortex layers are formed through different division patterns in different species, and there is likely uncharacterized diversity beyond what is described here. In species with closed root apical meristem, cortex layers develop from specific initials: in monocots, the first cortical cell arises from asymmetric division of cortex‐endodermis‐epidermis initial daughter (CEEID) and in dicots from cortex‐endodermis initial daughter (CEID). In the dicot species tomato, multiple cortex layers are generated in an inward‐to‐outward sequence through the divisions of the CEIDs and their cortical progeny (Ron *et al*., 2013). Conversely, in the monocot species rice, layer formation follows an outward‐to‐inward pattern driven by divisions of the CEEIDs and their cortical progeny (Pauluzzi *et al*., 2012). The number of cortex layers is determined by tightly regulated formative divisions, orchestrated in many lineages by the GRAS‐family transcription factor (TF) pair SHORTROOT (SHR)‐SCARECROW (SCR) (Wu *et al*., 2014; Henry *et al*., 2017; Ortiz‐Ramírez *et al*., 2021; Manzano *et al*., 2025). In Arabidopsis, SHR movement outwards from vasculature also specifies the innermost cortical layer as the endodermis (Andersen & Drapek, 2024). In other angiosperms, *shr* mutations do not cause a complete loss of the endodermis identity, thus SHR's specification role may be shared with additional regulators (Henry *et al*., 2017; Shaar‐Moshe & Brady, 2023; Manzano *et al*., 2025). Furthermore, it remains unknown which signals, such as possibly SHR, convey the spatial or the relative positions of any of the other cortex layers, and how these signals in turn may regulate the layer‐dependent capacities.

Increasingly, cell types and their characteristics are inferred from their transcriptional profiles. Is the heterogeneity of the cortex layers also seen in their transcriptomes? With the advent of the single cell/nuclei sequencing methods, diverse species with diverse root cellular architectures are being sequenced. From this expanding catalogue of species, it is clear that the transcriptional profiles between cortex parenchyma layers are not uniform, even before the onset of the differentiation programs (Ortiz‐Ramírez *et al*., 2021; Cervantes‐Pérez *et al*., 2022; Frank *et al*., 2023; Guillotin *et al*., 2023; Sun *et al*., 2023; Wang *et al*., 2024). Additionally, gene expression profiles have been characterized for cortex layer‐specific differentiation programs (Shiono *et al*., 2014; Kajala *et al*., 2021; Reynoso *et al*., 2022; Cantó‐Pastor *et al*., 2024; Jo *et al*., 2025b). While the cortex layers are distinct transcriptionally, detailed understanding of what makes the layers different from each other is missing. What are their specifying signals, what is unique in their gene regulatory networks, and what controls their differentiation capacities?

## IV. Shifting ground: cortex differentiation trajectories and their plasticity

Different cortex layers have the potential to differentiate into various tissues with stress‐protective functions. Here, we will discuss four of these: endodermis, exodermis, multiseriate cortical sclerenchyma and aerenchyma (Fig. 2). We have recently reviewed their morphologies and physiological roles in depth (Jones *et al*., 2025), and here we focus on their regulation: developmental, environmental (plasticity/inducibility) and positioning within cortex.

**Fig. 3:**
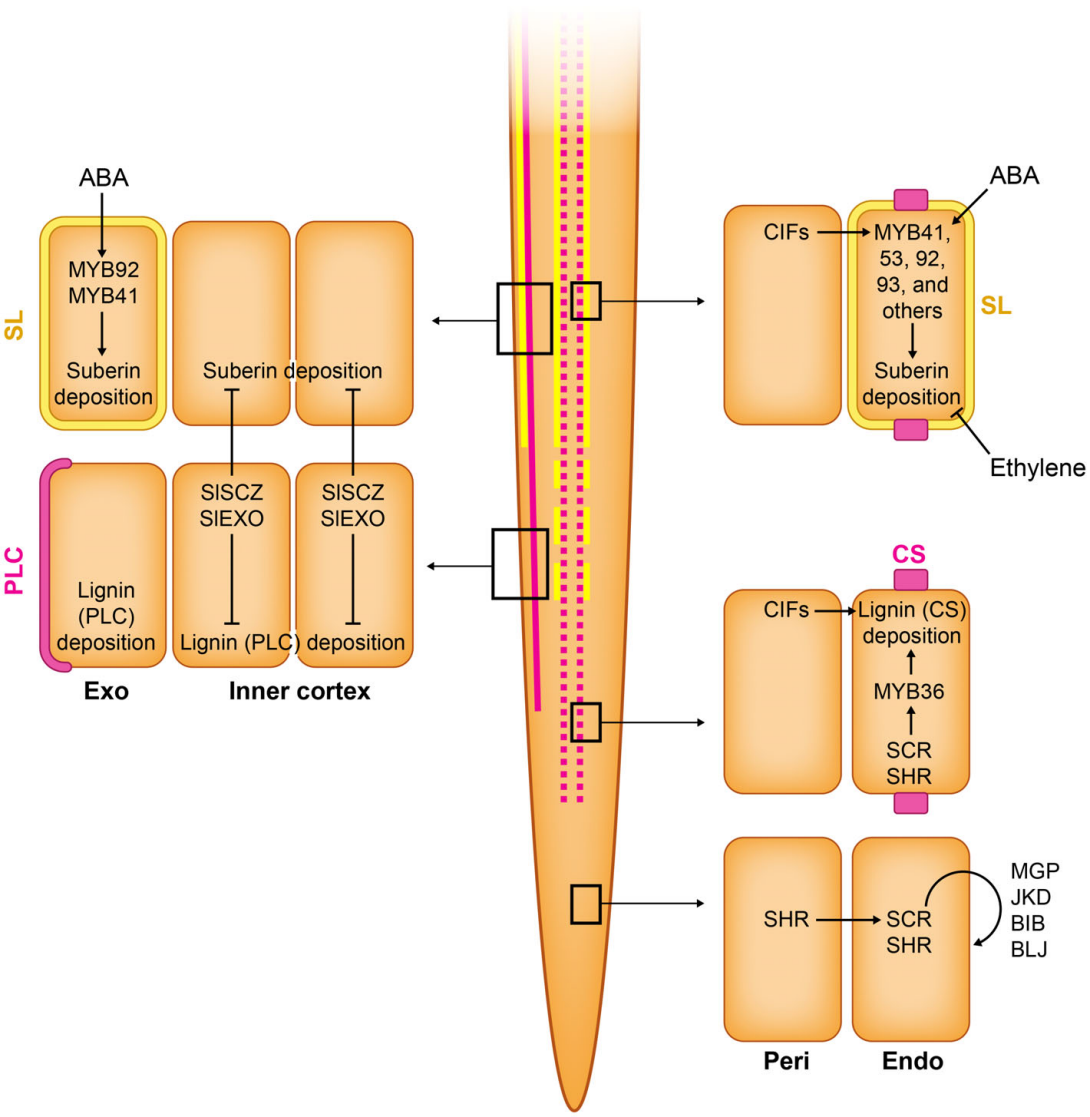
Regulators of endodermis and exodermis differentiation trajectories leading to lignin and suberin deposition into the cell wall. Exodermal suberin lamella (SL) is deposited into the cell walls in response to abscisic acid (ABA) in many species, and in tomato and chickpea, this has been shown to be regulated via homologs of suberin clade MYBs such as MYB92 and MYB41 (Cantó‐Pastor *et al*., 2024; Jo *et al*., 2025b). In tomato, SlSCZ and SlEXO restrict the deposition of lignin polar cap into the outermost cortex layer (exodermis) while inhibiting it in the inner cortex. SlSCZ and SlEXO also play a role in inhibiting suberin deposition in the inner cortex (Manzano *et al*., 2025). Endodermis in Arabidopsis is specified by the movement of SHR TF from the stele into the first cortex layer, where it activates and interacts with SCR TF. Note: SCR is redundant with BIRD family TFs (MAGPIE, MGP; JACKDAW, JKD; BALD‐IBIS, BIB; BLUEJAY, BLJ) who also help sequester SHR into the endodermis (Long *et al*., 2015). SHR–SCR pair activates MYB36, which is the master regulator of the Casparian strip development. Here, lignin gets deposited in an accurately specified anticlinal position, dividing the apoplast into vascular‐facing and soil‐facing (positioning mechanism not shown). Later in the developmental trajectory of endodermis, the cell walls also become suberized. This is a highly plastic and reversible trajectory as a multitude of environmental signals feed into it via auxin and ethylene. Master regulators of the suberin biosynthesis and deposition pathway are MYBs from the suberin clades (MYB41, 53, 92, 93, and others) who act redundantly. CIF, CASPARIAN STRIP INTEGRITY FACTOR; CS, Casparian strip; EXO, exodermis; ENDO, endodermis PERI, pericycle; PLC, Polar Lignin Cap; SCR, SCARECROW; SHR, SHORTROOT; SL, suberin lamella.

The two barrier cell types, endodermis and exodermis, are found in the innermost and the outermost cortex layers, respectively. Endodermis is found in all vascular plants, whereas exodermis is found in a large majority of flowering plants. As physical barriers, they play roles in protection against soil‐based stresses, such as drought, flooding, salinity, pests and pathogens (Kawa & Brady, 2022; Liu & Kreszies, 2023; Andersen & Drapek, 2024). The barrier capacities arise from lignin and suberin deposited in the cell walls of these barrier cell types. The differentiation programs for lignin and suberin deposition are largely distinct from each other (Fig. 3) (Andersen & Drapek, 2024; Cantó‐Pastor *et al*., 2024; Manzano *et al*., 2025). In addition, genetic program for suberin deposition differs even between the endodermal cell files, allowing inhibition of one of the programs to position the nonsuberized passage cells (Kraska *et al*., 2025).

For the endodermal barriers, there is a wealth of information from the model species Arabidopsis. The endodermal lignin deposition program is largely under developmental control, regulated via MYB36, a TF from the Myeloblastosis family, that is activated in endodermis by the SHR–SCR pair (Kamiya *et al*., 2015; Liberman *et al*., 2015). In addition to the SHR movement from vasculature that signals the innermost cortex layer to have the barrier capacities, the diffusion of CIF peptides (CASPARIAN STRIP INTEGRITY FACTOR) from vasculature into the apoplast serves also both as a positional cue and a surveillance system for functional barriers (Doblas *et al*., 2017; Nakayama *et al*., 2017). Together, SHR, MYB36 and CIF are sufficient to establish Casparian strip *de novo* (Drapek *et al*., 2018; Li *et al*., 2018). By contrast, the endodermal suberization in Arabidopsis is highly plastic in response to a range of environmental cues, such as drought, nutrient availability and soil microbiome as signaled via plant hormones abscisic acid (ABA) and ethylene (Kosma *et al*., 2014; Barberon *et al*., 2016; Salas‐González *et al*., 2021). Both ABA and CIF signals converge into suberin‐regulating MYB TFs, including MYB41, MYB53, MYB92 and MYB93 (Shukla *et al*., 2021), but what restricts their expression to specifically endodermis has not been shown. Developmental age and radial position of endodermis also affect suberization, and this may be regulated via the MYB68 expression domain (Kraska *et al*., 2025). Finally, suberization is a unique cortex modification, as it is reversible. In endodermis, ethylene signaling leads to degradation of suberin (Barberon *et al*., 2016), which likely serves as a mechanism protecting from potential trade‐offs, such as restriction of nutrient uptake, possibly through the action of suberin‐degrading GDSL‐type esterase/lipase proteins.

The understanding of regulation of exodermal barriers is only starting to emerge due to expanding model organisms for root development. Exodermis shows diversity in compositions and plasticity across angiosperms, including uniseriate and multiseriate forms (Liu & Kreszies, 2023). The differentiation programs for both suberin and lignin deposition can be under environmental regulation, for example, induced by ABA (Jo *et al*., 2025b). The exodermal suberin‐related differentiation program resembles that of Arabidopsis endodermis, as it is induced by ABA via MYB92 and MYB41 homologs in tomato (Cantó‐Pastor *et al*., 2024; Jo *et al*., 2025a) and chickpea (Jo *et al*., 2025b), whereas the exodermal lignin‐related differentiation program does not share regulators with the endodermis (Manzano *et al*., 2025). Finally, no cue has been identified that specifies the outermost cortex layer(s) as an exodermis, but in tomato, two TFs, SlEXO1 and SlSCZ, repress the lignification program and partially the suberization program in the inner cortex layers (Manzano *et al*., 2025), serving as a mechanism to separate the cortex layer capacities between exodermal and nonexodermal. In future, comparing closely related species with multiseriate and uniseriate exodermis could offer clues on how the exodermis capacity is restricted to specific layers.

Another cortical modification that uses lignin to strengthen the cell walls is multiseriate cortical sclerenchyma (MCS). This multi‐layered modification is seen in the outer cortex layers in specific root types, such as the adventitious roots, of many grass species (Schneider *et al*., 2021) and functions in increasing the tensile strength of the root, which supports root growth through compacted soil. Regulation of the MCS trajectory varies across maize cultivars (Fig. 1), and it can be under not only developmental (root type) but also environmental control, for example, induced by mechanostimulation (McCahill *et al*., 2025). The regulatory cues that launch the MCS trajectory are unknown, as are the cues that restrict it to the outer cortex. The first clues for the molecular mechanisms come from *Brachypodium distachyon*, where SECONDARY WALL NAC7 and gibberellic acid integrate the mechanostimulation cue into the already‐specified MCS cells and induce additional lignification (McCahill *et al*., 2025).

Aerenchyma, cortex modification established by formation of air spaces, helps gas diffusion in waterlogged soils, reduces metabolic cost of the root and prevents progression of pests and pathogens (Jones *et al*., 2025). Similar to barrier formation, aerenchyma can be regulated developmentally (constitutive) or environmentally (inducible), and it can form in different ways: through cell death (lysigenous), cell division and expansion (expansigenous) or cell separation (schizogenous). To date, molecular regulators are only known for the lysigenous aerenchyma. Constitutive lysigenous aerenchyma has been shown to be regulated by auxin and TF LBD1‐8 in rice (Yamauchi *et al*., 2019) and TF bHLH121 in maize (Schneider *et al*., 2023). The inducible lysigenous aerenchyma is often triggered by waterlogging in grasses, through signaling events including the ethylene accumulation, Ca^2+^ signaling, reactive oxygen species (ROS) and nitric oxide (Wany *et al*., 2017; Yamauchi *et al*., 2017) and in maize, also requires the bHLH121 (Schneider *et al*., 2023). In rice, the development of lysigenous aerenchyma and ROS accumulation starts from the outer cortex layers (Yamauchi *et al*., 2017), but it is not known what the underlying mechanisms are to position the aerenchyma initiation.

## V. Common ground: cortex layer capacities for symbiotic interactions

Cortex is also the site for symbiotic interactions, such those with nitrogen‐fixing rhizobia hosted inside nodules and with arbuscular mycorrhizal fungi (AMF). The capacity for these interactions is species‐ and cortex‐layer‐specific.

The main plant clade forming nitrogen‐fixing nodules is the legumes, and they form them in two main types: determinate nodules initiating in the outer cortex and indeterminate nodules initiating in the pericycle and the inner cortex. What specifies the distinct nodulation capacity of outer vs inner cortex is still emerging. The SHORTROOT‐SCARECROW pair is needed in cortex for nodulation competence for both indeterminate (Dong *et al*., 2021) and determinate nodules (Wang *et al*., 2022). Thus, the SHORTROOT‐SCARECROW tandem may serve as a cortex‐identity signal. In *Medicago truncatula*, the indeterminate nodule primordia initiate in the pericycle with cell‐type‐specific auxin biosynthesis (Xiao *et al*., 2025). This auxin is then transported specifically into the adjacent inner cortex where it serves as the cortex‐layer‐specific cue. Similarly for determinate nodule initiation in *Glycine max*, dynamic auxin transport in outer cortex layers is required (Gao *et al*., 2021). Hence, the suggested reason for the different nodulation capacities of cortex layers between lineages (or conditions) is their auxin sensitivities (Velandia *et al*., 2022), potentially adjusted by cortex‐layer‐specific deposition of flavonoids that inhibit auxin transport (Gao *et al*., 2021). The cortex‐layer‐specific regulation of flavonoid biosynthesis remains unknown. Moreover, functional Casparian strip is required for nodulation in *Lotus japonicus* (Shen *et al*., 2025).

In many species, the symbiotic structures with AMF, arbuscules, are formed in the inner cortex to be easily connected to the vasculature. Similar to nodulation, understanding of this specificity (or preferentiality) is still emerging, and the suggested vascular‐derived positional cues include sucrose and DELLAs, the GRAS‐family transcriptional regulators (Delaux & Gutjahr, 2024). The next step would be to dissect how these cues interact with the inner cortex gene regulatory network to capacitate it for the transcriptional programs of arbuscule formation.

## VI. Breaking ground: future directions to utilize cortex potential

As our knowledge of cortex specification, functions and plasticity increases, we stand at the point of building an integrated understanding of cortex. To do so, we need to map the relationship between cortex modifications. The suberized and/or lignified endodermis and exodermis, MCS and aerenchyma can exist next to each other or form successively in the same root (Klein *et al*., 2020). However, we still need to quantitatively characterize this coexistence and dissect its regulation by environment and root type. Does perturbing one cortex modification influence the others? Are there any functional redundancies or compensation mechanisms between cortex modifications? For example, does the development of lignified MCS affect the levels of exodermal lignification? Holistic assessment of cortex in diversity panels can address this question. The relationship between cortical barriers should also be elucidated from the perspective of their regulatory mechanisms. For example, it is known that suberization in both endodermis and exodermis is induced by ABA, but in many species, this response is observed in only either endodermis or exodermis (Cantó‐Pastor *et al*., 2024; Jo *et al*., 2025b). It remains unknown whether the differentiation trajectories across the cortex layers rely on the shared or distinct regulatory pathways. The existence of regulatory mechanisms connecting differentiation across cortex layers could balance the potential trade‐offs.

To investigate cortex modifications in combination, several emerging approaches are promising. First, to generate combinations of cortex modifications in uniform genetic background, microbes can be of help. The soil microbiome has a strong impact on several cortex‐related traits (number of cortex layers, endodermal suberization and lignification, aerenchyma) in sorghum roots (Kawa *et al*., 2024). While the causal isolates have only been so far identified for the increase in endodermal suberization (Kawa *et al*., 2024), further identification of isolates inducing other cortex modifications could lead to systematically ‘adding up’ individual modifications for combinatorial study. Second, spatial transcriptomics approaches can help link the transcriptomic heterogeneity to positional information across cortex layers. Finally, modeling cortex modifications can integrate quantitative cortex data to assess the contributions of spatially and temporally dynamic cortex modifications in heterogenous root systems comprised of different root types. The root hydraulics model, *MECHA*, allows exploring additive effects of cortex modifications on radial root conductivity (Couvreur *et al*., 2018). This modeling approach combined with the computational tool GRANAR demonstrated that radial water transport though the root differs between maize root types, as well as along the axis of each root (Heymans *et al*., 2021). Still, many cortex modifications or their physiological functions are not yet parameterized in the models, including MCS or root barriers on pathogen progression.

## VII. Conclusions

The cortex is dynamic and multifaceted, has distinct developmental trajectories, stress‐responsive capacities, and symbiotic competencies. As we deepen our understanding of the underlying regulatory networks, integrating developmental, environmental and spatial information will be essential. By exploring how different cortex modifications interact and contribute to plant function, we can uncover strategies to enhance plant resilience.

## Competing interests

None declared.

## Disclaimer

The New Phytologist Foundation remains neutral with regard to jurisdictional claims in maps and in any institutional affiliations.

## References

[nph70581-bib-0001] Andersen TG , Drapek C . 2024. The endodermis 1. In: Plant roots. Boca Raton, FL, USA: CRC Press, 90–103.

[nph70581-bib-0002] Barberon M , Vermeer JEM , De Bellis D , Wang P , Naseer S , Andersen TG , Humbel BM , Nawrath C , Takano J , Salt DE *et al*. 2016. Adaptation of root function by nutrient‐induced plasticity of endodermal differentiation. Cell 164: 447–459. 26777403 10.1016/j.cell.2015.12.021

[nph70581-bib-0003] Baum SF , Dubrovsky JG , Rost TL . 2002. Apical organization and maturation of the cortex and vascular cylinder in *Arabidopsis thaliana* (Brassicaceae) roots. American Journal of Botany 89: 908–920. 21665690 10.3732/ajb.89.6.908

[nph70581-bib-0004] Bower FO . 1935. Primitive land plants‐also known as the Archegoniatae. London, UK: MacMillan and Co.

[nph70581-bib-0005] Cantó‐Pastor A , Kajala K , Shaar‐Moshe L , Manzano C , Timilsena P , De Bellis D , Gray S , Holbein J , Yang H , Mohammad S *et al*. 2024. A suberized exodermis is required for tomato drought tolerance. Nature Plants 10: 118–130. 38168610 10.1038/s41477-023-01567-x PMC10808073

[nph70581-bib-1000] Cantó‐Pastor A , Manzano C , Brady SM . 2025. A way to interact with the world: complex and diverse spatiotemporal cell wall thickenings in plant roots. Annual Review of Plant Biology 76: 433–466. 10.1146/annurev-arplant-102820-112451 39745939

[nph70581-bib-0006] Cervantes‐Pérez SA , Thibivilliers S , Laffont C , Farmer AD , Frugier F , Libault M . 2022. Cell‐specific pathways recruited for symbiotic nodulation in the *Medicago truncatula* legume. Molecular Plant 15: 1868–1888. 36321199 10.1016/j.molp.2022.10.021

[nph70581-bib-0007] Couvreur V , Faget M , Lobet G , Javaux M , Chaumont F , Draye X . 2018. Going with the flow: multiscale insights into the composite nature of water transport in roots. Plant Physiology 178: 1689–1703. 30366980 10.1104/pp.18.01006 PMC6288756

[nph70581-bib-0008] Delaux P‐M , Gutjahr C . 2024. Evolution of small molecule‐mediated regulation of arbuscular mycorrhiza symbiosis. Philosophical Transactions of the Royal Society of London. Series B, Biological Sciences 379: 20230369. 39343030 10.1098/rstb.2023.0369 PMC11439497

[nph70581-bib-0009] Doblas VG , Smakowska‐Luzan E , Fujita S , Alassimone J , Barberon M , Madalinski M , Belkhadir Y , Geldner N . 2017. Root diffusion barrier control by a vasculature‐derived peptide binding to the SGN3 receptor. Science 355: 280–284. 28104888 10.1126/science.aaj1562

[nph70581-bib-0010] Dong W , Zhu Y , Chang H , Wang C , Yang J , Shi J , Gao J , Yang W , Lan L , Wang Y *et al*. 2021. An SHR‐SCR module specifies legume cortical cell fate to enable nodulation. Nature 589: 586–590. 33299183 10.1038/s41586-020-3016-z

[nph70581-bib-0011] Drapek C , Sparks EE , Marhavy P , Taylor I , Andersen TG , Hennacy JH , Geldner N , Benfey PN . 2018. Minimum requirements for changing and maintaining endodermis cell identity in the Arabidopsis root. Nature Plants 4: 586–595. 30061749 10.1038/s41477-018-0213-y PMC6135099

[nph70581-bib-0012] Esau K . 1967. Plant anatomy, 2^nd^ edn. New York, NY, USA: John Wiley & Sons.

[nph70581-bib-0013] Frank M , Fechete LI , Tedeschi F , Nadzieja M , Nørgaard MMM , Montiel J , Andersen KR , Schierup MH , Reid D , Andersen SU . 2023. Single‐cell analysis identifies genes facilitating rhizobium infection in *Lotus japonicus* . Nature Communications 14: 7171. 10.1038/s41467-023-42911-1 PMC10630511 37935666

[nph70581-bib-0014] Gao Z , Chen Z , Cui Y , Ke M , Xu H , Xu Q , Chen J , Li Y , Huang L , Zhao H *et al*. 2021. GmPIN‐dependent polar auxin transport is involved in soybean nodule development. Plant Cell 33: 2981–3003. 34240197 10.1093/plcell/koab183 PMC8462816

[nph70581-bib-0015] Guillotin B , Rahni R , Passalacqua M , Mohammed MA , Xu X , Raju SK , Ramírez CO , Jackson D , Groen SC , Gillis J *et al*. 2023. A pan‐grass transcriptome reveals patterns of cellular divergence in crops. Nature 617: 785–791. 37165193 10.1038/s41586-023-06053-0 PMC10657638

[nph70581-bib-0016] Henry S , Dievart A , Divol F , Pauluzzi G , Meynard D , Swarup R , Wu S , Gallagher KL , Périn C . 2017. SHR overexpression induces the formation of supernumerary cell layers with cortex cell identity in rice. Developmental Biology 425: 1–7. 28263767 10.1016/j.ydbio.2017.03.001

[nph70581-bib-0017] Heymans A , Couvreur V , Lobet G . 2021. Combining cross‐section images and modeling tools to create high‐resolution root system hydraulic atlases in *Zea mays* . Plant Direct 5: e334. 34355112 10.1002/pld3.334 PMC8320656

[nph70581-bib-0018] Iyer‐Pascuzzi AS , Jackson T , Cui H , Petricka JJ , Busch W , Tsukagoshi H , Benfey PN . 2011. Cell identity regulators link development and stress responses in the Arabidopsis root. Developmental Cell 21: 770–782. 22014526 10.1016/j.devcel.2011.09.009 PMC3204215

[nph70581-bib-0019] Jo L , Buti S , Artur MAS , Kluck RMC , Cantó‐Pastor A , Brady SM , Kajala K . 2025a. Transcription factors SlMYB41, SlMYB92 and SlWRKY71 regulate gene expression in the tomato exodermis. Journal of Experimental Botany eraf161. 40243161 10.1093/jxb/eraf161 PMC12646149

[nph70581-bib-0020] Jo L , Kluck RMC , Buti S , Holbein J , Chhillar H , Ding P , Franke R , Kajala K . 2025b. ABA‐induced MYB transcriptional module regulates the differentiation trajectory of chickpea exodermis. *bioRxiv*. doi: 10.1101/2025.01.03.631192.

[nph70581-bib-0021] Jones DH , Kajala K , Kawa D , Lopez‐Valdivia I , Kreszies T , Schneider HM . 2025. The root cortex of the Poaceae: a diverse, dynamic, and dispensable tissue. Plant and Soil. doi: 10.1007/s11104-025-07498-0. PMC12546384 41143299

[nph70581-bib-0022] Kajala K , Gouran M , Shaar‐Moshe L , Mason GA , Rodriguez‐Medina J , Kawa D , Pauluzzi G , Reynoso M , Canto‐Pastor A , Manzano C *et al*. 2021. Innovation, conservation, and repurposing of gene function in root cell type development. Cell 184: 5070. 34534466 10.1016/j.cell.2021.08.032

[nph70581-bib-0023] Kamiya T , Borghi M , Wang P , Danku JMC , Kalmbach L , Hosmani PS , Naseer S , Fujiwara T , Geldner N , Salt DE . 2015. The MYB36 transcription factor orchestrates Casparian strip formation. Proceedings of the National Academy of Sciences, USA 112: 10533–10538. 10.1073/pnas.1507691112 PMC4547244 26124109

[nph70581-bib-0024] Kawa D , Brady SM . 2022. Root cell types as an interface for biotic interactions. Trends in Plant Science 27: 1173–1186. 35792025 10.1016/j.tplants.2022.06.003

[nph70581-bib-0025] Kawa D , Thiombiano B , Shimels MZ , Taylor T , Walmsley A , Vahldick HE , Rybka D , Leite MFA , Musa Z , Bucksch A *et al*. 2024. The soil microbiome modulates the sorghum root metabolome and cellular traits with a concomitant reduction of Striga infection. Cell Reports 43: 113971. 38537644 10.1016/j.celrep.2024.113971 PMC11063626

[nph70581-bib-0026] Klein SP , Schneider HM , Perkins AC , Brown KM , Lynch JP . 2020. Multiple integrated root phenotypes are associated with improved drought tolerance. Plant Physiology 183: 1011–1025. 32332090 10.1104/pp.20.00211 PMC7333687

[nph70581-bib-0027] Kosma DK , Murmu J , Razeq FM , Santos P , Bourgault R , Molina I , Rowland O . 2014. AtMYB41 activates ectopic suberin synthesis and assembly in multiple plant species and cell types. The Plant Journal 80: 216–229. 25060192 10.1111/tpj.12624 PMC4321041

[nph70581-bib-0028] Kraska L , Melia JM , Nakano RT , Molina D , Formosa‐Jordan P , Ragni L , Andersen TG . 2025. MYB68 regulates radial endodermal differentiation and suberin patterning. Cell Reports 44: 115794. 40482030 10.1016/j.celrep.2025.115794

[nph70581-bib-0029] Kundu A , Moraes TA , Price RJ , Harrison RJ , Oldroyd GED . 2025. Getting to the route: the evolution of nitrogen‐fixing nodules. Annual Review of Cell and Developmental Biology. doi: 10.1146/annurev-cellbio-101123-093247. 40478967

[nph70581-bib-0030] Li P , Yu Q , Gu X , Xu C , Qi S , Wang H , Zhong F , Baskin TI , Rahman A , Wu S . 2018. Construction of a functional casparian strip in non‐endodermal lineages is orchestrated by two parallel signaling systems in *Arabidopsis thaliana* . Current Biology 28: 2777–2786. 30057307 10.1016/j.cub.2018.07.028

[nph70581-bib-0031] Liberman LM , Sparks EE , Moreno‐Risueno MA , Petricka JJ , Benfey PN . 2015. MYB36 regulates the transition from proliferation to differentiation in the Arabidopsis root. Proceedings of the National Academy of Sciences, USA 112: 12099–12104. 10.1073/pnas.1515576112 PMC4593085 26371322

[nph70581-bib-0032] Liu T , Kreszies T . 2023. The exodermis: a forgotten but promising apoplastic barrier. Journal of Plant Physiology 290: 154118. 37871477 10.1016/j.jplph.2023.154118

[nph70581-bib-0033] Long Y , Smet W , Cruz‐Ramírez A , Castelijns B , de Jonge W , Mähönen AP , Bouchet BP , Perez GS , Akhmanova A , Scheres B *et al*. 2015. Arabidopsis BIRD zinc finger proteins jointly stabilize tissue boundaries by confining the cell fate regulator SHORT‐ROOT and contributing to fate specification. Plant Cell 27: 1185–1199. 25829440 10.1105/tpc.114.132407 PMC4558684

[nph70581-bib-0034] Lux A , Luxová M , Abe J , Morita S . 2004. Root cortex: structural and functional variability and responses to environmental stress. Root Research 13: 117–131.

[nph70581-bib-0035] Lux A , Morita S , Abe J , Ito K . 2005. An improved method for clearing and staining free‐hand sections and whole‐mount samples. Annals of Botany 96: 989–996. 16192293 10.1093/aob/mci266 PMC4247103

[nph70581-bib-0036] Manzano C , Morimoto KW , Shaar‐Moshe L , Mason GA , Cantó‐Pastor A , Gouran M , De Bellis D , Ursache R , Kajala K , Sinha N *et al*. 2025. Regulation and function of a polarly localized lignin barrier in the exodermis. Nature Plants 11: 118–130. 39623209 10.1038/s41477-024-01864-z PMC11757151

[nph70581-bib-0037] McCahill IW , Abushal LT , Khahani B , Probert CF , Flockhart EL , Gregory GA , Li EZ , Zhang Y , Baumgart LA , O'Malley RC *et al*. 2025. Shoring up the base: the development and regulation of cortical sclerenchyma in grass nodal roots. Plant Physiology kiaf215. 40460244 10.1093/plphys/kiaf215

[nph70581-bib-0038] Nakayama T , Shinohara H , Tanaka M , Baba K , Ogawa‐Ohnishi M , Matsubayashi Y . 2017. A peptide hormone required for Casparian strip diffusion barrier formation in Arabidopsis roots. Science 355: 284–286. 28104889 10.1126/science.aai9057

[nph70581-bib-0039] Ortiz‐Ramírez C , Guillotin B , Xu X , Rahni R , Zhang S , Yan Z , Coqueiro Dias Araujo P , Demesa‐Arevalo E , Lee L , Van Eck J *et al*. 2021. Ground tissue circuitry regulates organ complexity in maize and Setaria. Science 374: 1247–1252. 34855479 10.1126/science.abj2327 PMC8719420

[nph70581-bib-0040] Pauluzzi G , Divol F , Puig J , Guiderdoni E , Dievart A , Périn C . 2012. Surfing along the root ground tissue gene network. Developmental Biology 365: 14–22. 22349629 10.1016/j.ydbio.2012.02.007

[nph70581-bib-2000] Perumalla CJ , Peterson CA , Enstone DE . 1990. A survey of angiosperm species to detect hypodermal Casparian bands. I. Roots with a uniseriate hypodermis and epidermis. Botanical Journal of the Linnean Society. Linnean Society of London 103: 93–112.

[nph70581-bib-3000] Peterson CA , Perumalla CJ . 1990. A survey of angiosperm species to detect hypodermal Casparian bands. II. Roots with a multiseriate hypodermis or epidermis. Botanical Journal of the Linnean Society. Linnean Society of London 103: 113–125.

[nph70581-bib-0041] Reynoso MA , Borowsky AT , Pauluzzi GC , Yeung E , Zhang J , Formentin E , Velasco J , Cabanlit S , Duvenjian C , Prior MJ *et al*. 2022. Gene regulatory networks shape developmental plasticity of root cell types under water extremes in rice. Developmental Cell 57: 1177–1192.e6. 35504287 10.1016/j.devcel.2022.04.013

[nph70581-bib-0042] Rich‐Griffin C , Eichmann R , Reitz MU , Hermann S , Woolley‐Allen K , Brown PE , Wiwatdirekkul K , Esteban E , Pasha A , Kogel K‐H *et al*. 2020. Regulation of cell type‐specific immunity networks in Arabidopsis roots. Plant Cell 32: 2742–2762. 32699170 10.1105/tpc.20.00154 PMC7474276

[nph70581-bib-0043] Ron M , Dorrity MW , de Lucas M , Toal T , Hernandez RI , Little SA , Maloof JN , Kliebenstein DJ , Brady SM . 2013. Identification of novel loci regulating interspecific variation in root morphology and cellular development in tomato. Plant Physiology 162: 755–768. 23575417 10.1104/pp.113.217802 PMC3668068

[nph70581-bib-0044] Salas‐González I , Reyt G , Flis P , Custódio V , Gopaulchan D , Bakhoum N , Dew TP , Suresh K , Franke RB , Dangl JL *et al*. 2021. Coordination between microbiota and root endodermis supports plant mineral nutrient homeostasis. Science 371: eabd0695. 33214288 10.1126/science.abd0695

[nph70581-bib-0045] Schiessl K , Jhu M‐Y . 2025. From roots to nodules: regulation of organogenesis in nitrogen‐fixing symbiosis. Current Opinion in Plant Biology 86: 102755. 40582138 10.1016/j.pbi.2025.102755

[nph70581-bib-0046] Schneider HM , Lor VS , Zhang X , Saengwilai P , Hanlon MT , Klein SP , Davis JL , Borkar AN , Depew CL , Bennett MJ *et al*. 2023. Transcription factor bHLH121 regulates root cortical aerenchyma formation in maize. Proceedings of the National Academy of Sciences, USA 120: e2219668120. 10.1073/pnas.2219668120 PMC10041174 36927156

[nph70581-bib-0047] Schneider HM , Strock CF , Hanlon MT , Vanhees DJ , Perkins AC , Ajmera IB , Sidhu JS , Mooney SJ , Brown KM , Lynch JP . 2021. Multiseriate cortical sclerenchyma enhance root penetration in compacted soils. Proceedings of the National Academy of Sciences, USA 118: e2012087118. 10.1073/pnas.2012087118 PMC8017984 33536333

[nph70581-bib-0048] Shaar‐Moshe L , Brady SM . 2023. SHORT‐ROOT and SCARECROW homologs regulate patterning of diverse cell types within and between species. New Phytologist 237: 1542–1549. 36457304 10.1111/nph.18654

[nph70581-bib-0049] Shen D , Micic N , Venado RE , Bjarnholt N , Crocoll C , Persson DP , Samwald S , Kopriva S , Westhoff P , Metzger S *et al*. 2025. Apoplastic barriers are essential for nodule formation and nitrogen fixation in *Lotus japonicus* . Science 387: 1281–1286. 40112074 10.1126/science.ado8680

[nph70581-bib-0050] Shiono K , Yamauchi T , Yamazaki S , Mohanty B , Malik AI , Nagamura Y , Nishizawa NK , Tsutsumi N , Colmer TD , Nakazono M . 2014. Microarray analysis of laser‐microdissected tissues indicates the biosynthesis of suberin in the outer part of roots during formation of a barrier to radial oxygen loss in rice (*Oryza sativa*). Journal of Experimental Botany 65: 4795–4806. 24913626 10.1093/jxb/eru235

[nph70581-bib-0051] Shukla V , Han J‐P , Cléard F , Lefebvre‐Legendre L , Gully K , Flis P , Berhin A , Andersen TG , Salt DE , Nawrath C *et al*. 2021. Suberin plasticity to developmental and exogenous cues is regulated by a set of MYB transcription factors. Proceedings of the National Academy of Sciences, USA 118: e2101730118. 10.1073/pnas.2101730118 PMC8488582 34551972

[nph70581-bib-0052] Shulse CN , Cole BJ , Ciobanu D , Lin J , Yoshinaga Y , Gouran M , Turco GM , Zhu Y , O'Malley RC , Brady SM *et al*. 2019. High‐throughput single‐cell transcriptome profiling of plant cell types. Cell Reports 27: 2241–2247. 31091459 10.1016/j.celrep.2019.04.054 PMC6758921

[nph70581-bib-0053] Sun Z , Jiang S , Wang D , Li L , Liu B , Ran Q , Hu L , Xiong J , Tang Y , Gu X *et al*. 2023. Single‐cell RNA‐seq of *Lotus japonicus* provide insights into identification and function of root cell types of legume. Journal of Integrative Plant Biology 65: 1147–1152. 36537698 10.1111/jipb.13435

[nph70581-bib-0054] Velandia K , Reid JB , Foo E . 2022. Right time, right place: the dynamic role of hormones in rhizobial infection and nodulation of legumes. Plant Communications 3: 100327. 35605199 10.1016/j.xplc.2022.100327 PMC9482984

[nph70581-bib-0055] Wang C , Li M , Zhao Y , Liang N , Li H , Li P , Yang L , Xu M , Bian X , Wang M *et al*. 2022. SHORT‐ROOT paralogs mediate feedforward regulation of D‐type cyclin to promote nodule formation in soybean. Proceedings of the National Academy of Sciences, USA 119: e2108641119. 10.1073/pnas.2108641119 PMC8784155 35022232

[nph70581-bib-0056] Wang G , Ryu KH , Dinneny A , Carlson J , Goodstein D , Lee J , Oh D‐H , Oliva M , Lister R , Dinneny JR *et al*. 2024. Diversification of gene expression across extremophytes and stress‐sensitive species in the Brassicaceae. *bioRxiv*. doi: 10.1101/2024.06.21.599952.

[nph70581-bib-0057] Wang Y , Huan Q , Li K , Qian W . 2021. Single‐cell transcriptome atlas of the leaf and root of rice seedlings. Journal of Genetics and Genomics 48: 881–898. 34340913 10.1016/j.jgg.2021.06.001

[nph70581-bib-0058] Wany A , Kumari A , Gupta KJ . 2017. Nitric oxide is essential for the development of aerenchyma in wheat roots under hypoxic stress. Plant, Cell & Environment 40: 3002–3017. 10.1111/pce.13061 28857271

[nph70581-bib-0059] Wu S , Lee C‐M , Hayashi T , Price S , Divol F , Henry S , Pauluzzi G , Perin C , Gallagher KL . 2014. A plausible mechanism, based upon Short‐Root movement, for regulating the number of cortex cell layers in roots. Proceedings of the National Academy of Sciences, USA 111: 16184–16189. 10.1073/pnas.1407371111 PMC4234584 25352666

[nph70581-bib-0060] Xiao TT , Müller S , Shen D , Liu J , Adema K , van Seters A , Franssen H , Bisseling T , Kulikova O , Kohlen W . 2025. Nodule organogenesis in *Medicago truncatula* requires local stage‐specific auxin biosynthesis and transport. Plant Physiology 197: kiaf133. 40181792 10.1093/plphys/kiaf133 PMC12002018

[nph70581-bib-0061] Yamauchi T , Tanaka A , Inahashi H , Nishizawa NK , Tsutsumi N , Inukai Y , Nakazono M . 2019. Fine control of aerenchyma and lateral root development through AUX/IAA‐ and ARF‐dependent auxin signaling. Proceedings of the National Academy of Sciences, USA 116: 20770–20775. 10.1073/pnas.1907181116 PMC6789968 31548376

[nph70581-bib-0062] Yamauchi T , Yoshioka M , Fukazawa A , Mori H , Nishizawa NK , Tsutsumi N , Yoshioka H , Nakazono M . 2017. An NADPH oxidase RBOH functions in rice roots during lysigenous aerenchyma formation under oxygen‐deficient conditions. Plant Cell 29: 775–790. 28351990 10.1105/tpc.16.00976 PMC5435434

